# Hygiene of the skin: when is clean too clean?

**DOI:** 10.3201/eid0702.010215

**Published:** 2001

**Authors:** E Larson

**Affiliations:** Columbia University School of Nursing, New York, New York, USA. ell23@columbia.edu

## Abstract

Skin hygiene, particularly of the hands, is a primary mechanism for reducing contact and fecal-oral transmission of infectious agents. Widespread use of antimicrobial products has prompted concern about emergence of resistance to antiseptics and damage to the skin barrier associated with frequent washing. This article reviews evidence for the relationship between skin hygiene and infection, the effects of washing on skin integrity, and recommendations for skin care practices.


### Hygiene of the Skin: When Is Clean Too Clean?

**Elaine Larson**
Columbia University School of Nursing, New York, New York, USA

Skin hygiene, particularly of the hands, is a primary mechanism for reducing contact and fecal-oral transmission of infectious agents. Widespread use of antimicrobial products has prompted concern about emergence of resistance to antiseptics and damage to the skin barrier associated with frequent washing. This article reviews evidence for the relationship between skin hygiene and infection, the effects of washing on skin integrity, and recommendations for skin care practices.

For over a century, skin hygiene, particularly of the hands, has been accepted as a primary mechanism to control the spread of infectious agents. Although the causal link between contaminated hands and infectious disease transmission is one of the best-documented phenomena in clinical science, several factors have recently prompted a reassessment of skin hygiene and its effective practice.

In industrialized countries, exposure to potential infectious risks has increased because of changing sociologic patterns (e.g., more frequent consumption of commercially prepared food and expanded child-care services). Environmental sanitation and public health services, despite room for improvement, are generally good. In addition, choices of hygienic skin care products have never been more numerous, and the public has increasing access to health- and product-related information (1). This paper reviews evidence for the relationship between skin hygiene and infection, the effects of washing on skin integrity, and recommendations for skin care practices for the public and health-care professionals.

### Does Skin Cleansing Reduce Risk for Infection?

### Personal Bathing and Washing

There is a clear temporal relationship between improvement in general levels of cleanliness in society and improved health. Greene (2) used historical and cross-cultural evidence and causal inference to associate personal hygiene with better health. However, the role of personal cleanliness in the control of infectious diseases over the past century is difficult to measure, since other factors have changed at the same time (e.g., improved public services, waste disposal, water supply, commercial food handling, and nutrition) (3).

Studies of personal and domestic hygiene and its relationship to diarrhea in developing countries demonstrate the effectiveness of proper waste disposal, general sanitary conditions, and handwashing (4,5). However, aside from hand cleansing, specific evidence is lacking to link bathing or general skin cleansing with preventing infections. Part of the difficulty in demonstrating a causal association between general bathing or skin care and gastrointestinal infection is that interventions to reduce diarrheal disease have been multifaceted, often including health education, improved waste disposal, decontaminating the water supply, and general improvement in household sanitation as well as personal hygiene (6,7). Risk for diarrheal disease has also been linked to the level of parental education (8). Multiple influences complicate definition of the impact of any single intervention.

In 11 studies reviewed by Keswick et al. (9), use of antimicrobial soaps was associated with substantial reductions in rates of superficial cutaneous infections. Another 15 experimental studies demonstrated a reduction in bacteria on the skin with use of antimicrobial soaps, but none assessed rates of infection as an outcome.

Extensive studies of showering and bathing conducted since the 1960s demonstrated that these activities increase dispersal of skin bacteria into the air and ambient environment (10-12), probably through breaking up and spreading of microcolonies on the skin surface and resultant contamination of surrounding squamous cells. These studies prompted a change in practice among surgical personnel, who are now generally discouraged from showering immediately before entering the operating room. Other investigators have shown that the skin microflora varies between persons but is remarkably consistent for each person over time. Even without bathing for many days, the flora remain qualitatively and quantitatively stable (13-15).

For surgical or other high-risk patients, showering with antiseptic agents has been tested for its effect on postoperative wound infection rates. Such agents, unlike plain soaps, reduce microbial counts on the skin (16-18). In some studies, antiseptic preoperative showers or baths have been associated with reduced postoperative infection rates, but in others, no differences were observed (19-21). Whole-body washing with chlorhexidine-containing detergent has been shown to reduce infections among neonates (22), but concerns about absorption and safety preclude this as a routine practice. Several studies have demonstrated substantial reductions in rates of acquisition of methicillin-resistant *Staphylococcus aureus* in surgical patients bathed with a triclosan-containing product (23,24). Hence, preoperative showering or bathing with an antiseptic may be justifiable in selected patient populations.

### Hand Hygiene for the General Public

Much contemporary evidence for a causal link between handwashing and risk for infection in community settings comes from industrialized countries (5,7,25-27). Although

Address for correspondence: Elaine Larson, Columbia University School of Nursing, 630 W. 168th St., New York, NY 10032, USA; fax: 212-305-0722; e-mail: ell23@columbia.edu many of these studies may be limited by confounding by other variables, evidence of an important role for handwashing in preventing infections is among the strongest available for any factor studied. Reviews of studies linking handwashing and reduced risk for infection have been recently published (28,29). The most convincing evidence of the benefits of handwashing for the general public is for prevention of infectious agents found transiently on hands or spread by the fecal-oral route or from the respiratory tract (30). Plain soaps are considered adequate for this purpose.

Several highly publicized, serious outbreaks from commercially prepared foods have raised questions about food safety and the hygienic practices of food handlers and others in the service professions. Despite public awareness, however, handwashing generally does not meet recommended standards—members of the public wash too infrequently and for short periods of time (31).

These factors have led to suggestions that antimicrobial products should be more universally used, and a myriad of antimicrobial soaps and skin care products have become commercially available. While antimicrobial drug-containing products are superior to plain soaps for reducing both transient pathogens and colonizing flora, widespread use of these agents has raised concerns about the emergence of bacterial strains resistant to antiseptic ingredients such as triclosan (32,33). Such resistance has been noted in England and Japan (34), and molecular mechanisms for the development of resistance have been proposed (32,35). Although in some settings exposure to antiseptics has occurred for years without the appearance of resistance, a recent study described mutants of *Escherichia coli* selected for resistance to one disinfectant that were also multiply-antibiotic resistant (35). Some evidence indicates that long-term use of topical antimicrobial agents may alter skin flora (36,37). The question remains whether antimicrobial soaps provide sufficient benefit in reducing transmission of infection without added risk or cost.

### Hand Hygiene in Health-Care Settings

Issues regarding hand hygiene practices among health-care professionals have been widely discussed and may be even more complicated than those in the general public. Unless patient care involves invasive procedures or extensive contact with blood and body fluids, current guidelines recommend plain soap for handwashing (38,39); however, infection rates in adult or neonatal intensive care units or surgery may be further reduced when antiseptic products are used (40-42).

### Skin Barrier Properties and Effect of Hand Hygiene Practices

The average adult has a skin area of about 1.75 $m^2$. The superficial part of the skin, the epidermis, has five layers. The stratum corneum, the outermost layer, is composed of flattened dead cells (corneocytes or squames) attached to each other to form a tough, horny layer of keratin mixed with several lipids, which help maintain the hydration, pliability, and barrier effectiveness of the skin. This horny layer has been compared to a wall of bricks (corneocytes) and mortar (lipids) and serves as the primary protective barrier (43). Approximately 15 layers make up the stratum corneum, which is completely replaced every 2 weeks; a new layer is formed approximately daily (44). From healthy skin, approximately $10^7$ particles are disseminated into the air each day, and 10% of these skin squames contain viable bacteria (45). The dispersal of organisms is greater in males than in females and varies between persons using the same hygienic regimen by as much as fivefold (46).

Water content, humidity, pH, intracellular lipids, and rates of shedding help retain the protective barrier properties of the skin. When the barrier is compromised (e.g., by hand hygiene practices such as scrubbing), skin dryness, irritation, cracking, and other problems may result. Although the palmar surface of the hand has twice as many cell layers and the cells are >30 times thicker than on the rest of the skin (47), palms are quite permeable to water (48).

Long-term changes in skin pH associated with handwashing may pose a concern since some of the antibacterial characteristics of skin are associated with its normally acidic pH (49). In one report, pH increased 0.6 to 1.8 units after handwashing with plain soap for 1 to 2 min and then gradually declined to baseline levels over a period of 45 min to 2 hr (50). Some soaps can be associated with long-standing changes in skin pH, reduction in fatty acids, and subsequent changes in resident flora such as propionibacter (51).

In an investigation of the effect on skin of repeated use of two washing agents, all skin function tests (stratum corneum capacitative resistance, lipids, transepidermal water loss, pH, laser Doppler flow, and skin reddening) were markedly changed after a single wash, and after 1 week further damage was noted (52). In a study of irritant skin reactions induced by three surfactants, damage lasted for several days; complete skin repair was not achieved for 17 days (53).

Soaps and detergents have been described as the most damaging of all substances routinely applied to skin (43). Anionic and cationic detergents are more harmful than nonionic detergents (54), and increased concentrations of surfactant result in more rapid, severe damage (55). Each time the skin is washed, it undergoes profound changes, most of them transient. However, among persons in occupations such as health care in which frequent handwashing is required, long-term changes in the skin can result in chronic damage, irritant contact dermatitis and eczema, and concomitant changes in flora.

Irritant contact dermatitis, which is associated with frequent handwashing, is an occupational risks for health-care professionals, with a prevalence of 10% to 45% (56-58). The prevalence of damaged skin on the hands of 410 nurses was reported to be 25.9% in one survey, with 85.6% of nurses reported to have problems at some time. Skin damage was correlated with frequency of glove use and handwashing (56). Washing with plain soap may actually increase the potential for microbial transmission because of a 17-fold increase in the dispersal of bacterial colonies from the skin of the hands (59). Skin condition clearly plays a major role in risk for transmission.

### Microbiology of Hands of Health-Care Professionals

Damaged skin more often harbors increased numbers of pathogens. Moreover, washing damaged skin is less effective at reducing numbers of bacteria than washing normal skin, and numbers of organisms shed from damaged skin are often higher than from healthy skin (60,61). The microbial flora on the clean hands of nurses (samples taken immediately after handwashing) have been reported in several recent studies (Table). Methicillin resistance among coagulase-negative

Table. Microbial flora colonizing hands of health-care professionals

A. Microbial counts

| Year (ref.) | Sample (No. subjects) | Mean $log_{10}$ CFU |
|---|---|---|
| 1986 (62) | Staff of bone marrow transplant unit (22) | 4. 89 |
| 1992 (63) | Pediatric staff, Peru (62) | 5.88 |
| 1997 (64) | Nurses in acute care unit (40) | 5.61 |

B. Resistance of coagulase-negative staphylococcal flora

| Year (ref.) | Sample (No. isolates) | Resistant (%) to methicillin | Resistant (%) to tetracycline |
|---|---|---|---|
| 1986 (62) | Staff of bone marrow transplant unit (50) | 68.0 | 23.0 |
| 1988 (65) | Oncology, dermatology staff (152) | 50.7 | 30.7 |
| 1992 (63) | Pediatric staff, Peru (279) | 40.9 | 45.4 |
| 1997 (64) | Acute care nurses (122) | 59.0 | 10.5 |

staphylococcal flora on hands did not seem to increase during the 1980s to the 1990s, and tetracycline resistance decreased (Table).

### When Is Clean Too Clean?

Even with use of antiseptic preparations, which substantially reduce counts of hand flora, no reductions beyond an equilibrium level are attained (66). The numbers of organisms spread from the hands of nurses who washed frequently with an antimicrobial soap actually increased after a period of time; this increase is associated with declining skin health (67). In a recent survey, nurses with damaged hands were twice as likely to be colonized with *S. hominis, S. aureus*, gram-negative bacteria, enterococci, and *Candida* spp. and had a greater number of species colonizing the hands (64).

The trend in both the general public and among health-care professionals toward more frequent washing with detergents, soaps, and antimicrobial ingredients needs careful reassessment in light of the damage done to skin and resultant increased risk for harboring and transmitting infectious agents. More washing and scrubbing are unlikely to be better and may, in fact, be worse. The goal should be to identify skin hygiene practices that provide adequate protection from transmission of infecting agents while minimizing the risk for changing the ecology and health of the skin and increasing resistance in the skin flora.

### Recommendations for the General Public

Bathing or showering cleans the skin by mechanical removal of bacteria shed on corneocytes. Bacterial counts are at least as high or higher after bathing or showering with a regular soap than before. Frequent bathing has aesthetic and stress-relieving benefits but serves little microbiologic purpose. Mild, nonantimicrobial soap should suffice for routine bathing. Bathing with an antimicrobial product reduces rates of cutaneous infection and could be beneficial when skin infections are likely or before certain surgical procedures. With those exceptions, available data do not support a recommendation for bathing with antimicrobial products.

No single recommendation for hand hygiene practices in the general population would be adequate. The potential advantage of sustained antimicrobial activity for certain occupations (e.g., food handlers and child-care providers) must be balanced with the theoretical possibility of emergence of resistant strains and perhaps other, as yet unrecognized, safety issues.

An alternative to detergent-based antiseptic products is the use of alcohol hand rinses, which have recently become widely available over the counter. Their advantages include rapid and broad-spectrum activity, excellent microbicidal characteristics, and lack of potential for emergence of resistance. Alcohol-based products could be recommended for use among persons who need immediate protection after touching contaminated surfaces or before and after contact with someone at high risk for infection.

Since hands are a primary mode of fecal-oral and respiratory transmission, specific indications for use of antiseptic hand products by the general public are close physical contact with persons at high risk for infection (e.g., neonates, the very old, or immunosuppressed); close physical contact with infected persons; infection with an organism likely to be transmitted by direct contact (diarrhea, upper respiratory infection, skin infections); or work in a setting in which infectious disease transmission is likely (food preparation, crowded living quarters such as chronic-care residences, prisons, child-care centers, and preschools).

### Recommendations for the Health-Care Professional

### Detergent-Based Antiseptics or Alcohol

Because of increasingly vulnerable patient populations, the demand for hand hygiene among health-care professionals has never been greater. However, frequent handwashing is not only potentially damaging to skin, it is also time-consuming and expensive (68). Finnish investigators demonstrated that after frequent washing the hands of patient-care providers became damaged and posed greater risk to themselves and patients than if they had washed less often. A mild emulsion cleansing rather than handwashing with liquid soap was associated with a substantial improvement in the skin of nurses' hands (69). Alcohol-based formulations are superior to antiseptic detergents for rapid microbial killing on skin (66,67,70-72) and, with the addition of appropriate moisturizers, are probably milder (67,73,74). Since alcohols are rapid acting, are broad spectrum, and require no washing or drying, damage caused by detergents and mechanical friction from toweling is avoided.

### Use of Lotions and Moisturizers

Moisturizing is beneficial for skin health and reducing microbial dispersion from skin, regardless of whether the product used contains an antibacterial ingredient (75-77). Because of differences in the content and formulations of lotions and creams, products vary greatly in their effectiveness (78,79). Lotions used with products containing chlorhexidine gluconate must be carefully selected to avoid neutralization by anionic surfactants (80). The role of emollients and moisturizers in improving skin health and reducing microbial spread is an area for additional research.

To improve the skin condition of health-care professionals and reduce their chances of harboring and shedding microorganisms from the skin, the following measures are recommended: 1) For damaged skin, mild, nonantimicrobial skin cleansing products may be used to remove dirt and debris. If antimicrobial action is needed (e.g., before invasive procedures or handling of highly susceptible patients) a waterless, alcohol-based product may be used. 2) In clinical areas such as the operating room and neonatal and transplant units, shorter, less traumatic washing regimens may be used instead of lengthy scrub protocols with brushes or other harsh mechanical action. 3) Effective skin emollients or barrier creams may be used in skin-care regimens and procedures for staff (and possibly patients as well). 4) Skin moisturizing products should be carefully assessed for compatibility with any topical antimicrobial products being used and for physiologic effects on the skin (81).

### Conclusions

From the public health perspective, more frequent use of current hygiene practices may not necessarily be better (i.e., perhaps sometimes clean is "too clean"), and the same recommendations cannot be applied to all users or situations. Future investigation is likely to improve understanding of the interaction between skin physiology, microbiology, and ecology and the role of the skin in the transmission of infectious diseases.

Dr. Larson is professor of pharmaceutical and therapeutic research, The School of Nursing, and professor of epidemiology, Mailman School of Public Health, Columbia University. She is editor of the American Journal of Infection Control and former chair of the Healthcare Infection Control Practices Advisory Committee and member of CDC's National Center for Infectious Diseases Board of Scientific Counselors.

### References

1. Sattar SA, Tetro J, Springthorpe VS. Impact of changing societal trends on the spread of infections in American and Canadian homes. Am J Infect Control 1999;27:S4-S21.
2. Green VW. Cleanliness and the health revolution. New York: Soap and Detergent Association; 1984. Available from: URL:http://ww.sdahq.org/about/order_formjs.html
3. Larson E. Social and economic impact of infectious diseases—United States. Clin Performance and the Quality of Health Care 1997;5:31-7.
4. Ekanem EE, Akitoye CO, Adedeji OT. Food hygiene behaviour and childhood diarrhoea in Lagos, Nigeria: a case-control study. J Diarrhoeal Dis Res 1991;9:219-26.
5. Alam N, Wojtyniak B, Henry FJ, Rahaman MM. Mothers' personal and domestic hygiene and diarrhoea incidence in young children in rural Bangladesh. Int J Epidemiol 1989;18:242-7.
6. Feachem RG. Interventions for the control of diarrhoeal diseases among young children: promotion of personal and domestic hygiene. Bull World Health Organ 1984;62:467-76.
7. Haggerty PA, Muladi K, Kirkwood BR, Ashworth A, Manunebo M. Community-based hygiene education to reduce diarrhoeal disease in rural Zaire: impact of the intervention on diarrhoeal morbidity. Int J Epidemiol 1994;23:1050-9.
8. Manun'ebo MN, Haggerty PA, Kalengaie M, Ashworth A, Kirkwood BR. Influence of demographic, socioeconomic and environmental variables on childhood diarrhoea in a rural area of Zaire. J Trop Med Hyg 1994;97:31-8.
9. Keswick BH, Berge CA, Bartolo RG, Watson DD. Antimicrobial soaps: their role in personal hygiene. In: Aly R, Beutner KR, Maibach H, editors. Cutaneous infection and therapy. New York: Marcel Dekker, Inc.; 1997. p. 49-82.
10. Speers R, Bernard H, O'Grady F, Shooter RA. Increased dispersal of skin bacteria into the air after shower-baths. Lancet 1965;1:478-83.
11. Hall GS, Mackintosh CA, Hoffman PN. The dispersal of bacteria and skin scales from the body after showering and after application of a skin lotion. J Hyg (Camb) 1986;97:289-98.
12. Ulrich JA. Dynamics of bacterial skin populations. In: Maibach HI, Hildick-Smith G, editors. Skin bacteria and their role in infection. New York: McGraw-Hill; 1965. p. 219-34.
13. Evans CA. Persistent individual differences in the bacterial flora of the skin of the forehead: numbers of propionibacteria. J Invest Dermatol 1975;64:42-6.
14. Leyden JJ, McGinley KJ, Nordstrom KM, Webster GF. Skin microflora. J Invest Dermatol 1987;88:65s-72.
15. Hartmann AA. Daily bath and its effect on the normal human skin flora quantitative: and qualitative investigations of the aerobic skin flora. Arch Dermatol Res 1979;265:153-64.
16. Paulson DS. Efficacy evaluation of a 4% chlorhexidine gluconate as a full-body shower wash. Am J Infect Control 1993;21:205-9.
17. Kaiser AB, Kernodle DS, Barg NL, Petracek MR. Influence of preoperative showers on staphylococcal skin colonization: a comparative trial of antiseptic skin cleansers. Ann Thorac Surg 1988;45:35-8.
18. Byrne DJ, Napier A, Cuschieri A. Rationalizing whole body disinfection. J Hosp Infect 1990;15:183-7.
19. Mackenzie I. Preoperative skin preparation and surgical outcome. J Hosp Infect 1988;11(Suppl B):27-32.
20. Rotter ML, Larsen SO, Cooke EM, Dankert J, Daschner F, Greco D, et al. A comparison of the effects of preoperative whole-body bathing with detergent alone and with detergent containing chlorhexidine glucontate on the frequency of wound infections after clean surgery. J Hosp Infect 1988;11:310-20.
21. Ayliffe GAJ, Noy MF, Babb JR, Davies JG, Jackson J. A comparison of pre-operative bathing with chlorhexidine-detergent and non-medicated soap in the prevention of wound infection. J Hosp Infect 1983;4:237-44.
22. Meberg A, Schoyen R. Bacterial colonization and neonatal infections. Effects of skin and umbilical disinfection in the nursery. Acta Paediatr Scand 1985;74:366-71.
23. Tuffnell DJ, Croton RS, Hemingway DM, Hartley MN, Wake PN, Garvey RJ. Methicillin resistant *Staphylococcus aureus*; the role of antisepsis in the control of an outbreak. J Hosp Infect 1987;10:255-9.
24. Bartzokas CA, Paton JH, Gibson MF, Graham F, McLoughlin GA, Croton RS. control and eradication of methicillin-resistant *Staphylococcus aureus* on a surgical unit. N Engl J Med 1984;311:1422-5.
25. Sempertegui F, Estrella B, Correa E, Aguirre L, Saa B, Torres M, et al. Risk of diarrheal disease in Ecuadorian day-care centers. Pediatr Infect Dis 1995;14:606-12.
26. Shahid NS, Greenough WB, Samadi AR, Huq MI, Rahman N. Hand washing with soap reduces diarrhoea and spread of bacterial pathogens in a Bangladesh village. J Diarrhoeal Dis Res 1996;14:85-9.
27. Rudland S, Little M, Kemp P, Miller A, Hodge J. The enemy within: diarrheal rates among British and Australian troops in Iraq. Mil Med 1996;161:728-31.
28. Larson E. A causal link between hand washing and risk of infection? Examination of the evidence. Infect Control Hosp Epidemiol 1988;9:28-36.
29. Bryan JL, Cohran J, Larson EL. Hand washing: a ritual revisited. Crit Care Nurs Clin North Am 1995;7:617-26.
30. Gwaltney JM, Moskalski PB, Hendley JO. Hand-to-hand transmission of rhinovirus colds. Ann Intern Med 1978;88:463-7.
31. ASM inagurates nationwide public education effort. ASM News 1996;62:547-8.

32. Russell AD, Hammond SA, Morgan JR. Bacterial resistance to antiseptics and disinfectants. J Hosp Infect 1986;7:213-25.
33. APIC position statement. The use of antimicrobial household products. APIC News 1997;(Nov/Dec):13.
34. Sasatsu M, Shimizu K, Noguchi N, Kong M. Triclosan-resistant *Staphylococcus aureus* [letter]. Lancet 1993;342:248.
35. Moken MC, McMurry LM, Levy SB. Selection of multiple-antibiotic-resistant (mar) mutants of *Escherichia coli* by using the disinfectant pine oil: roles of the mar and acrAB loci. Antimicrob Agents Chemother 1997;41:2770-2.
36. Ehrenkranz NJ, Taplin D, Butt P. Antibiotic-resistant bacteria on the nose and skin: colonization and cross-infection. Proceedings from Sixth Interscience Conference on Antimicrobial Agents and Chemotherapy. Philadelphia: American Society for Microbiology. Antimicrob Agents Chemother; 1966. p. 255-64.
37. Bruun JN, Solberg CO. Hand carriage of gram negative bacilli and *Staphylococcus aureus*. BMJ 1973;2:580-2.
38. Hospital Infection Control Practices Advisory Committee. Guideline for isolation precautions in hospitals. Am J Infect Control 1996;24:24-52.
39. Larson E, the 1992, 1993, and 1994 APIC Guideline Committees. APIC guideline for handwashing and hand antisepsis in health care settings. Am J Infect Control 1995;23:251-69.
40. Doebbeling BN, Stanley GL, Sheetz CT, Pfaller MA, Houston AK, Annis L, et al. Comparative efficacy of alternative handwashing agents in reducing nosocomial infections in intensive care units. N Engl J Med 1992;327:88-93.
41. Zafar AB, Butler RC, Reese DJ, Gaydos LA, Mennonna PA. Use of 0.3% triclosan (Bacti-Stat*) to eradicate an outbreak of methicillin-resistant *Staphylococcus aureus* in a neonatal nursery. Am J Infect Control 1995;23:200-8.
42. Webster J, Faoagali JL, Cartwright D. Elimination of methicillin-resistant *Staphylococcus aureus* from a neonatal intensive care unit after hand washing with triclosan. J Paediatr Child Health 1994;30:59-64.
43. Jarrett A, editor. The physiology and pathophysiology of the skin. New York: Academic Press; 1978.
44. Schaefer H, Redelmeier TE. Skin barrier: principles of percutaneous absorption. Basel: Karger; 1996.
45. Noble WC, Davies RR. Studies on the dispersal of staphylococci. J Clin Pathol 1965;18:16-20.
46. Noble WC. Dispersal of skin microorganisms. Br J Dermatol 1975;93:477-85.
47. Holbrook KA, Odland GF. Regional differences in the thickness (cell layers) of the human stratum: an ultra-structural analysis. J Invest Dermatol 1974;62:415.
48. Blank IH. Factors which influence the water content of the stratum corneum. J Invest Dermatol 1952;18:433.
49. Maki DG. The use of antiseptics for handwashing by medical personnel. J Chemother 1989;1(Suppl):3-11.
50. Klauder JV, Gross BA. Actual causes of certain occupational dermatoses. III. a further study with special reference to effect of alkali on the skin, effect of soap on pH of skin, modern cutaneous detergents. Arch Dermatol Symp 1951;63:1-23.
51. Hoffler U, Gloor M, Peters G, Ko HL, Brautigam A, Thurn A, et al. Qualitative and quantitative investigations on the resident bacterial skin flora in healthy persons and in the non-affected skin of patients with seborrheic eczema. Arch Dermatol Res 1980;268:297-312.
52. Grunewald AM, Gloor M, Gehring W, Kleesz P. Damage to the skin by repetitive washing. Contact Dermatitis 1995;32:225-32.
53. Wilhelm KP, Freitag G, Wolff HH. Surfactant-induced skin irritation and skin repair. Evaluation of the acute human irritation model by noninvasive techniques. J Am Acad Dermatol 1994;30:944-9.
54. Dugard PH, Scheuplein RJ. Effect of ionic surfactants on the permeability of human epidermis: an electrometric study. J Invest Dermatol 1973;60:263-5.
55. Scheuplein RJ, Ross L. Effects of surfactants and solvents on the permeability of epidermis. J Soc Cosmetol Chem 1970;21:853-6.
56. Larson E, Friedman C, Cohran J, Treston-Aurand J, Green S. Prevalence and correlates of skin damage on hands of nurses. Heart Lung 1997;26:404-12.
57. Sproat LJ, Uveges RE. Epidemiology of hand dermatitis in dental personnel. Mil Med 1995;160:335-8.
58. Stingeni L, Lapomarda V, Lisi P. Occupational hand dermatitis in hospital environments. Contact Dermatitis 1995;33:172-6.
59. Meers PD, Yeo GA. Shedding of bacteria and skin squames after handwashing. J Hyg (Camb) 1978;81:99-105.
60. Ojajarvi J. Effectiveness of hand washing and disinfection methods in removing transient bacteria after patient nursing. J Hyg (Camb) 1980;85:193-203.
61. Parry MF, Hutchinson JH, Brown NA, Wu CH, Estreller L. Gram-negative sepsis in neonates: a nursery outbreak due to hand carriage of *Citrobacter diversus*. Pediatrics 1980;65:1105-9.
62. Larson E, McGinley K, Grove G, Leyden J, Talbot G. Physiologic, microbiologic, and seasonal effects of handwashing on the skin of health care personnel. Am J Infect Control l986;l4:5l-9.
63. Larson E, McGinley K, Foglia A, Leyden J, Boland N, Larson J, et al. Handwashing practices and resistance and density of bacterial hand flora on two pediatric units in Lima, Peru. Am J Infect Control 1992;20:65-72.
64. Larson EL, Norton Hughes CA, Pyrek JD, Sparks SM, Cagatay EU, Bartkus JM. Changes in bacterial flora associated with skin damage on hands of health care personnel. Am J Infect Control 1998;26:513-21.
65. Horn W, Larson E, McGinley K, Leyden JJ. Microbial flora on the hands of health care personnel: differences in composition and antibacterial resistance. Infect Control Hosp Epidemiol 1988;9:189-93.
66. Lilly HA, Lowbury EJL, Wilkins MD. Limits to progressive reduction of resident skin bacteria by disinfection. J Clin Pathol 1979;32:382-5.
67. Ojajarvi J, Makela P, Rantsalo I. Failure of hand disinfection with frequent hand washing: a need for prolonged field studies. J Hyg (Camb) 1977;79:107-19.
68. Voss A, Widmer AF. No time for handwashing? Handwashing versus alcoholic rub: can we afford 100% compliance? Infect Control Hosp Epidemiol 1997;28:205-8.
69. Lauharanta J, Ojajarvi J, Sarna S, Makela P. Prevention of dryness and eczema of the hands of hospital staff by emulsion cleansing instead of washing with soap. J Hosp Infect 1991;17:207-15.
70. Morrison AJ, Gratz J, Cabzudo I, Wenzel RP. The efficacy of several new handwashing agents for removing non-transient bacterial flora from hands. Infect Control 1986;7:268-72.
71. Rotter ML, Koller W. Test models for hygienic handrub and hygienic handwash: the effects of two different contamination and sampling techniques. J Hosp Infect 1992;20;163-71.
72. Hobson DW, Woller W, Anderson L, Guthery E. Development and evaluation of a new alcohol-based surgical hand scrub with persistent antimicrobial characteristics and brushless application. Am J Infect Control 1998;26:507-12.
73. Larson E, Eke P, Laughon B. Efficacy of alcohol-based hand rinses under frequent use conditions. Antimicrob Agents Chemother 1986;30:542-4.
74. Larson E, Silberger M, Jakob K, Whittier S, Lai L, DellaLatta P, et al. Assessment of alternative hand hygiene regimens to improve skin health among neonatal ICU nurses. Heart Lung 2000;29:136-42.
75. Murray J, Calman RM. Control of cross-infection by means of an antiseptic hand cream. BMJ 1955;1:81-3.

76. Zelickson AS, Zelickson BD, Zelickson BM. Measurements by transmission electron microscopy of "dry" skin before and after application of a moisturizing cream. Am J Dermatopathol 1982;4:205-8.
77. Grunewald AM, Gloor M, Gehring W, Kleesz P. Efficacy of barrier creams. In: Elsner P, Maibach HI, editors. Irritant dermatitis: new clinical and experimental aspects. Curr Probl Dermatol 1995;23:187-97.
78. Loden M. Barrier recovery and influence of irritant stimuli in skin treated with a moisturinzing cream. Contact Dermatitis 1997;36:256-60.
79. Schluter-Wigger W, Elsner P. Efficacy of four commercially available protective creams in the repetitive irritation test (RIT). Contact Dermatitis 1996;34:278-83.
80. Frantz SW, Haines KA, Azar CG, Ward JI, Homan SM, Roberts RB. Chlorhexidine gluconate activity against clinical isolates of vancomycin-resistant *Enterococcus faecium* (VREF) and the effects of moisturizing agents on CGH residue accumulation on the skin. J Hosp Infect 1997;37:157-64.
81. Larson E. Skin hygiene and infection prevention: more of the same or different approaches? Clin Infect Dis 1999;29:1287-94.
